# Peripheral Administration of Tumor Necrosis Factor-Alpha Induces Neuroinflammation and Sickness but Not Depressive-Like Behavior in Mice

**DOI:** 10.1155/2015/716920

**Published:** 2015-07-28

**Authors:** Steven Biesmans, Jan A. Bouwknecht, Luc Ver Donck, Xavier Langlois, Paul D. Acton, Patrick De Haes, Nima Davoodi, Theo F. Meert, Niels Hellings, Rony Nuydens

**Affiliations:** ^1^BIOMED, Hasselt University, Agoralaan C Building, 3590 Diepenbeek, Belgium; ^2^Neurosciences, Janssen Research & Development, Division of Janssen Pharmaceutica NV, Turnhoutseweg 30, 2340 Beerse, Belgium; ^3^Molecular Imaging, Janssen Research & Development LLC, Welsh & McKean Roads, Spring House, PA 19477-0779, USA

## Abstract

Clinical observations indicate that activation of the TNF-*α* system may contribute to the development of inflammation-associated depression. Here, we tested the hypothesis that systemic upregulation of TNF-*α* induces neuroinflammation and behavioral changes relevant to depression. We report that a single intraperitoneal injection of TNF-*α* in mice increased serum and brain levels of the proinflammatory mediators TNF-*α*, IL-6, and MCP-1, in a dose- and time-dependent manner, but not IL-1*β*. Protein levels of the anti-inflammatory cytokine IL-10 increased in serum but not in the brain. The transient release of immune molecules was followed by glial cell activation as indicated by increased astrocyte activation in bioluminescent Gfap-luc mice and elevated immunoreactivity against the microglial marker Iba1 in the dentate gyrus of TNF-*α*-challenged mice. Additionally, TNF-*α*-injected mice were evaluated in a panel of behavioral tests commonly used to study sickness and depressive-like behavior in rodents. Our behavioral data imply that systemic administration of TNF-*α* induces a strong sickness response characterized by reduced locomotor activity, decreased fluid intake, and body weight loss. Depressive-like behavior could not be separated from sickness at any of the time points studied. Together, these results demonstrate that peripheral TNF-*α* affects the central nervous system at a neuroimmune and behavioral level.

## 1. Introduction

Clinical depression is a chronic, disabling psychiatric condition that affects over 120 million people worldwide [1]. It is predicted that, by 2030, depression will be the second leading cause of disability in the world [2]. Although its etiology remains poorly understood, it is generally accepted that depression is a multifactorial disorder with numerous interacting systems underlying its pathogenesis. A number of clinical observations suggest that dysregulation of the immune system might also play a role in the development of depression, at least in a subset of susceptible individuals. For example, depression frequently occurs as a comorbidity of medical conditions characterized by a chronic inflammatory component including rheumatoid arthritis [3], cancer [4], type 2 diabetes [5], stroke [6], obesity [7], and coronary artery disease [8]. Even in absence of other medical illnesses, depressed patients often show elevated circulating levels of inflammatory mediators such as proinflammatory cytokines and the acute-phase C-reactive protein [9, 10]. Moreover, up to half of cancer and hepatitis C patients that receive therapeutic administration of proinflammatory cytokines eventually develop depressive symptoms [11–13].

There are several indications that tumor necrosis factor-*α* (TNF-*α*) is one of the key cytokines involved in the pathogenesis of inflammation-associated depression. Recent meta-analyses confirmed that circulating levels of TNF-*α* are significantly higher in depressed patients compared with healthy control subjects [10, 14]. Moreover, elevated plasma levels of TNF-*α* are associated with treatment resistance to conventional antidepressants [15]. In hepatitis C patients that are chronically treated with interferon-*α*, increased blood levels of TNF-*α* correlate with the development of depressive symptoms [16]. Furthermore, peripheral administration of anti-TNF-*α* antibodies improves depressed mood in patients suffering from psoriasis [17], Crohn's disease [18], and rheumatoid arthritis [19]. TNF-*α* antagonism has also been shown to improve treatment resistant depression in a subgroup of patients with high baseline inflammatory biomarkers [20].

Inflammation-associated depression is often studied in rodents by peripheral administration of immunostimulants such as bacterial lipopolysaccharide (LPS). It is known that systemic injection of LPS elicits a widespread immune response, characterized by the release of numerous immune mediators and the occurrence of sickness, a behavioral state comprised of symptoms such as malaise, lethargy, decreased motor activity and appetite, sleep disturbances, and increased sensitivity to pain [21, 22]. There are some indications that this sickness response is followed by a phase of depressive-like behavior [23–25]. However, the characteristics of sickness can substantially confound the evaluation of depressive-like behavior in behavioral tests. For example, sick animals display reduced exploration, which can potentially interfere with measurements of immobility used to estimate behavioral despair in paradigms such as the forced swim (FST) and tail suspension test (TST) [26]. Moreover, sick animals eat and drink less, which can bias measures of sweetened fluid intake in assays designed to evaluate anhedonia (the inability to experience pleasure from naturally rewarding activities). Using a panel of behavioral paradigms in mice, we recently demonstrated that it is difficult to separate depressive-like behavior from sickness following acute peripheral LPS administration [27].

Based on the fact that systemic LPS administration induces a broad immune response and the clinical data linking TNF-*α* to human inflammation-associated depression, we hypothesized that peripheral administration of TNF-*α* itself may provide a more specific approach to study depressive-like behavior in mice. Indeed, systemic administration of TNF-*α* has already been shown to have central effects as indicated by increased proinflammatory gene expression in the brain and the development of sickness [28, 29]. Moreover, intracerebroventricular (i.c.v.) injection of TNF-*α* was shown to lead to depressive-like behavior in mice [30, 31]. However, to our knowledge, no study has systematically assessed the effect of peripheral TNF-*α* administration on neuroinflammation and depressive-like behavior over time. Therefore, the present series of experiments aimed at characterizing the central effects of systemic TNF-*α* injection by combining multiple techniques to quantify neuroinflammation and behavioral changes. First, serum and brain levels of immune mediators were quantified at several time points after systemic TNF-*α* administration. Next, transgenic bioluminescent Gfap-luc mice were used to evaluate the time course of TNF-*α*-induced astrocyte activation, as a marker of glial cell activation* in vivo*. Then, the occurrence of glial cell activation was confirmed by immunohistochemistry using the microglial marker ionized calcium-binding adapter molecule 1 (Iba1). Finally, TNF-*α*-injected mice were tested in a panel of behavioral paradigms to assess whether depressive-like behavior could be separated over time from sickness.

## 2. Material and Methods

### 2.1. Animals and TNF-*α*

All animal care and use were performed in accordance with the Guide for the Care and Use of Laboratory Animals (NRC) and experimental protocols were approved by the Institutional Ethical Committee on Animal Experimentation, according to applicable regional law. Male NMRI mice were purchased from Charles River Laboratories (France), male wild-type FVB mice were purchased from Janvier (France), and male transgenic Gfap-luc mice (FVB/N-Tg(Gfap-luc)-Xen) were obtained from Taconic Laboratories (USA). The latter animals express luciferase under the transcriptional control of the glial fibrillary acidic protein (Gfap) promoter [32] and are commonly used as a model system for noninvasive quantification of astrocyte activation in living animals over time [27, 33, 34]. Unless mentioned otherwise, animals were housed in groups of 4 per cage under a normal 12:12 h light-dark cycle (lights on at 06:00 a.m. with a 30 min dim and rise phase). Food and water were available ad libitum.

Recombinant mouse TNF-*α* was purchased from Biolegend (product ID 575208) and dissolved in sterile phosphate buffered saline prior to injection.

### 2.2. Cytokine Measurements

10-week-old male NMRI mice were injected intraperitoneally (i.p.) with 0, 63, 125, or 250 *μ*g/kg TNF-*α* (*n* = 6-7 per group) and sacrificed by decapitation at 2 h, 6 h, or 24 h. This dose range was based on results from the open field test (OFT) (see Section 2.5). Serum and whole brain samples were collected and processed as previously described [27]. Concentrations of interferon-*γ* (IFN-*γ*), interleukin- (IL-) 1*β*, IL-6, IL-10, monocyte chemoattractant protein-1 (MCP-1), and TNF-*α* were determined in each sample using a mouse cytokine/chemokine magnetic bead panel kit from Merck Millipore. All steps in the assay were conducted according to the manufacturer's instructions. Cytokine levels below detection limit were assigned a value equal to the lowest detectable value of that cytokine.

### 2.3. *In Vivo* Bioluminescence Imaging

Astrocyte activation in 10-week-old male Gfap-luc mice was quantified before (baseline) and at 2 h, 6 h, 24 h, 48 h, 72 h, and 96 h after i.p. administration of either 0, 63, or 250 *μ*g/kg TNF-*α* (*n* = 7 per group). Brain bioluminescence was detected as described previously [27, 35]. Briefly, Gfap-luc mice were anesthetized by inhalation of 2% isoflurane in 1 L/min oxygen, shaved on the head, and injected with 126 mg/kg D-luciferin (Promega, product ID E1601) in the tail vein. Three minutes later the animals were scanned with a charge-coupled device camera (IVIS Imaging System 200 Series, PerkinElmer) mounted on a dark box. Photon emission from the whole brain was measured using Living Image 3.2 software (PerkinElmer) in a region of interest (ROI) that was kept constant across mice. Bioluminescence coming from the ears was considered to be basal Gfap activity and was excluded from the ROI. Imaging signals were measured in physical units of surface radiance (photons/s/cm^2^/steradian [sr]).

### 2.4. Immunohistochemistry

10-week-old male FVB mice were injected i.p. with vehicle or 250 *μ*g/kg TNF-*α* (*n* = 8 per group) and tissue was collected 24 h later. Immunohistochemical staining of Iba1 protein in the dentate gyrus of the hippocampus was performed using a rabbit polyclonal anti-Iba1 primary antibody (1 : 500, Wako Chemicals) and a fluorescent Alexa 555 goat anti-rabbit secondary antibody (1 : 500, Invitrogen), as previously described [27].

### 2.5. Behavioral Tests

All behavioral tests were performed on separate groups of 10-week-old male NMRI mice. The OFT and FST setups were custom-made and were described in detail previously [27].

Two independent OFTs were performed in this study. In the first OFT, mice were injected with 0, 63, 250, or 1000 *μ*g/kg TNF-*α* (*n* = 10 per group) and repeatedly tested at 2 h, 6 h, 24 h, and 48 h after administration. Two of the 10 mice that received 1000 *μ*g/kg TNF-*α* died during the first 24 h after injection. Therefore, it was decided to take 250 *μ*g/kg TNF-*α* as the highest test dose for all experiments and to repeat the OFT with a vehicle and 125 *μ*g/kg TNF-*α* group.

In the FST, mice (*n* = 10 per group) were injected with 0, 63, 125, or 250 *μ*g/kg TNF-*α* and tested at 2 h, 6 h, 24 h, and 48 h after administration.

The sucrose preference test (SPT) started by single-housing the animals in individually ventilated cages (*L* × *W* × *H*: 35 × 31 × 16 cm; Tecniplast, Italy) fitted with two 250 mL drinking bottles and ad libitum access to food. Each bottle contained either filtered tap water or a 5% sucrose solution. The location of the bottles on the cage was randomized during every exposure session with half of the animals receiving sucrose on the left and half on the right. The SPT protocol lasted for 5 days and consisted of a familiarization and a test phase. The familiarization phase started on day 1 by exposing all mice to one water- and one 5% sucrose-filled bottle (W/S) for 24 h. On day 2, the animals had free access to two water-filled bottles (W/W) until 4:00 p.m., after which they were fluid-deprived overnight. The test phase started on day 3 by injecting mice i.p. with 0, 63, 125, or 250 *μ*g/kg TNF-*α* (*n* = 10 per group). To test the effects of TNF-*α* at 2 h, 6 h, 24 h, and 48 h, the animals were presented with W/S during a 1 h exposure period at these time points. Mice were fluid-deprived in between exposure periods. In order to avoid a protracted deprivation period between the 24 h and 48 h time points, mice were given access to W/W from 4:00 to 5:00 p.m. on day 4.

In the SPT study using a within-subject design it became clear that exposing the thirsty animals to W/S at 2 h affected the total volume intake at 6 h (less thirsty). To exclude that the effects of TNF-*α* were confounded by retesting the same animals over time, the SPT study was repeated in an independent between-subjects design study using separate groups of TNF-*α* challenged mice that were tested at either 6 h or 24 h. These mice underwent the same familiarization phase as described above. At the beginning of the test phase, the animals were injected with 0, 63, 125, or 250 *μ*g/kg i.p. TNF-*α* (*n* = 10 per group). At 6 h after TNF-*α*, mice from the 6 h time point group were exposed to W/S for a 1 h period. Animals from the 24 h time point were allowed to drink W/W for 1 h at 6 h after TNF-*α* in order to avoid a protracted deprivation period between TNF-*α* administration and the 24 h time point. At 24 h, mice from the 24 h group were presented with W/S during a 1 h exposure period.

In both SPT studies, the amount drunk by a mouse was determined by subtracting the weight of a bottle at the start of an exposure period and at the end (taking fluid density as 1 g/mL). Total fluid intake was calculated as the total change in volume from both bottles combined. A fluid intake that was greater than the mean +2x standard deviation was considered to be an invalid measure that probably resulted from leaking bottles. Invalid measures were replaced by the group mean of the relevant solution (water or sucrose). This occurred for less than 4% of all bottle measurements. Sucrose preference was calculated as the percentage of consumed sucrose solution of the total fluid intake.

### 2.6. Statistical Analysis

SPSS Statistics software version 20 (IBM Inc.) was used for data analysis. Analysis of variance (ANOVA) or repeated measures ANOVA (rmANOVA) was performed to determine the statistical significance of differences between treatment groups. To correct for potential violation of the sphericity assumption, a Greenhouse-Geisser correction epsilon (*ε*) was used for repeated measures analysis [36]. This correction multiplies both the numerator and the denominator degrees of freedom by *ε* and the significance of the *F*-ratio is evaluated with the new degrees of freedom, resulting in a more conservative statistical test. To account for the skewness of the data distribution, bioluminescence measurements and cytokine concentrations were log-transformed prior to analysis. ANOVAs and rmANOVAs were considered statistically significant if *P* < 0.05. When appropriate, post hoc comparisons were made by using an independent samples *t*-test with a Bonferroni-corrected *P* value. For consistency between the analysis and the visualization of bioluminescence measurements and cytokine concentrations, the group means and its standard error of the mean (SEM) were back-transformed and visually presented on a logarithmic scale. All other data are expressed as mean ± SEM on a linear scale.

## 3. Results

### 3.1. TNF-*α* Increases Immune Mediator Levels in Serum and Brain

**Figure 1:**
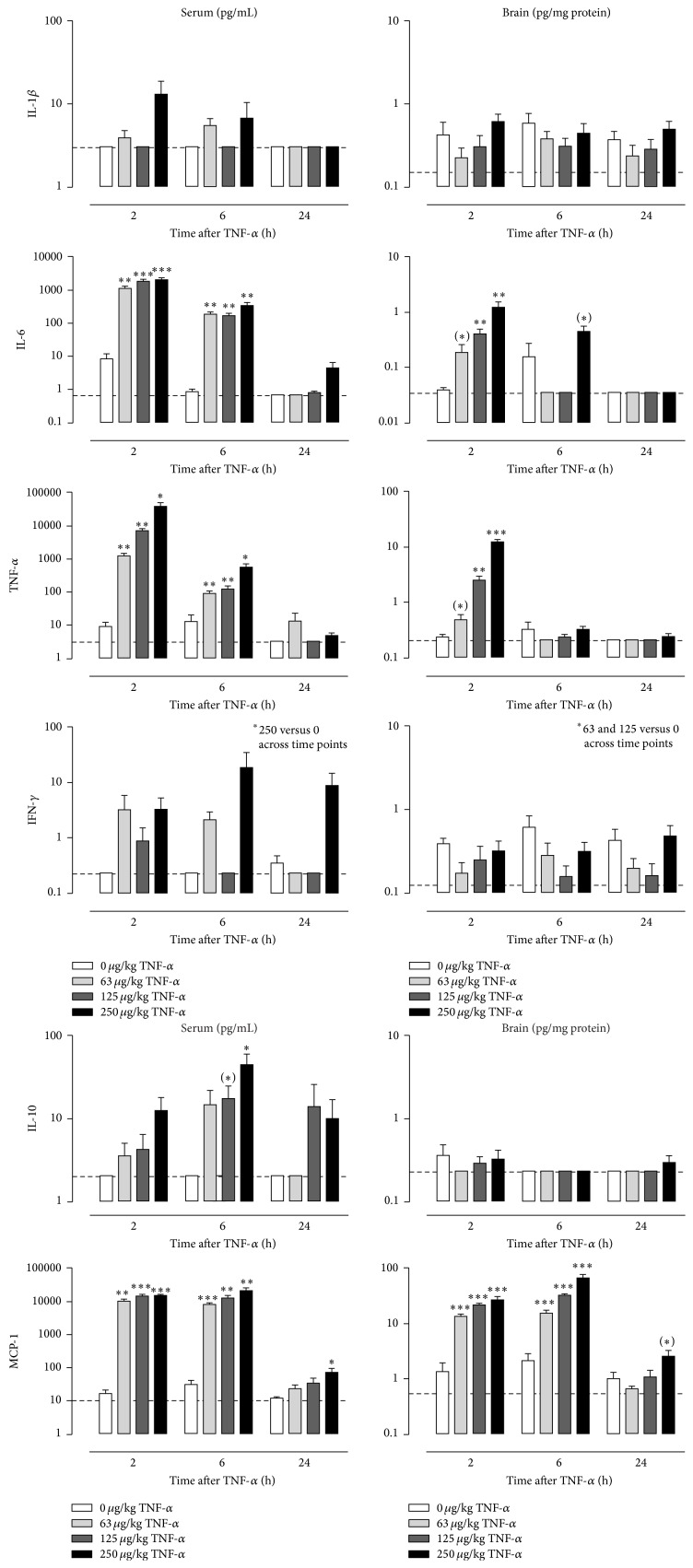
Peripheral TNF-*α* administration induces the release of immune mediators in serum and brain. Time course of serum (left) and brain concentrations (right) of interleukin- (IL-) 1*β*, IL-6, tumor necrosis factor-*α* (TNF-*α*), interferon-*γ* (IFN-*γ*), IL-10, and monocyte chemoattractant protein-1 (MCP-1) as measured at 2 h, 6 h, and 24 h after i.p. TNF-*α* injection. Note that serum concentrations are shown as pg/mL while brain levels are expressed in pg/mg protein. Dashed lines indicate the detection limit of the measured analyte. Graphs are plotted as mean + SEM (*n* = 6-7 per group). Data were analyzed by ANOVA followed by independent samples *t*-test. (∗) 0.1 < *P* < 0.05; ^∗^ *P* < 0.05; ^∗∗^ *P* < 0.01; ^∗∗∗^ *P* < 0.001 compared to 0 *μ*g/kg TNF-*α*.

To characterize the immunological response to peripheral TNF-*α* injection, serum and brain levels of several immune factors were quantified at 2 h, 6 h, and 24 h after administration. Factorial ANOVA showed a significant time × dose interaction on serum levels of IL-6, TNF-*α*, and MCP-1 (IL-6: *F*(6,68) = 13.4, *P* < 0.001; TNF-*α*: *F*(6,66) = 15.7, *P* < 0.001; MCP-1: *F*(6,68) = 7.2, *P* < 0.001), a main effect of time and dose on serum levels of IL-10 (time: *F*(2,68) = 5.3, *P* < 0.01; dose: *F*(3,68) = 4.8, *P* < 0.01), and a main effect of dose on serum levels of IL-1*β* (*F*(3,68) = 3.2, *P* < 0.05) and IFN-*γ* (*F*(3,68) = 5.8, *P* < 0.01). Post hoc analysis demonstrated that serum levels of IL-6, TNF-*α*, and MCP-1 peaked at 2 h after systemic injection of TNF-*α* and then gradually waned over time (Figure 1, left). The TNF-*α*-induced release of IL-10 followed a different time course as serum levels of this cytokine were only elevated at 6 h after TNF-*α*. At 24 h, the serum concentrations of IL-6, TNF-*α*, and IL-10 had returned to baseline values, while MCP-1 levels remained significantly elevated in mice that were injected with 250 *μ*g/kg TNF-*α*. Serum concentrations of IFN-*γ* were higher across time points in animals that received 250 *μ*g/kg TNF-*α*, while IL-1*β* in serum was not significantly different at the post hoc level.

For brain tissue, a significant time × dose interaction was found on protein levels of IL-6, TNF-*α*, and MCP-1 (IL-6: *F*(6,67) = 6.4, *P* < 0.001; TNF-*α*: *F*(6,67) = 70.2, *P* < 0.001; MCP-1: *F*(6,67) = 15.4, *P* < 0.001) and a main effect of dose on IFN-*γ* levels (*F*(3,67) = 4.0, *P* < 0.05). No significant effect of time or dose could be detected on brain levels of IL-1*β* or IL-10. Post hoc analysis revealed that brain levels of IL-6 and TNF-*α* peaked at 2 h and had dissipated by 6 h (Figure 1, right). However, at 6 h there was still a trend for elevated IL-6 levels in mice that had received 250 *μ*g/kg TNF-*α*. Comparable to the time course of its release in serum, brain levels of MCP-1 remained strongly elevated from 2 h until 6 h after treatment. At 24 h, there was still a trend for increased MCP-1 levels in mice injected with 250 *μ*g/kg TNF-*α*. Brain concentrations of IFN-*γ* were decreased across time points in animals from the 63 and 125 *μ*g/kg TNF-*α* group, but not in mice that received 250 *μ*g/kg TNF-*α* group.

### 3.2. TNF-*α* Induces Glial Cell Activation

**Figure 2:**
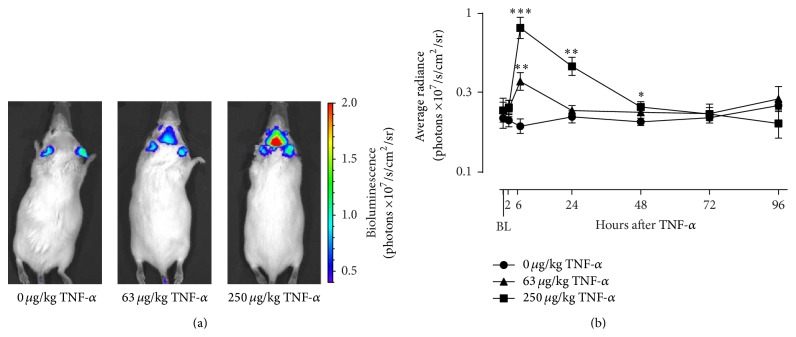
TNF-*α* activates astrocytes in a dose- and time-dependent manner. Intraperitoneal injection of TNF-*α* caused a clear bioluminescent signal in the brain of Gfap-luc mice, as shown in representative images taken at 6 h after injection (a). This signal peaked at 6 h and then gradually waned over time (b). The color scale indicates the number of photons emitted from the animal per second. The graph is plotted as mean ± SEM (*n* = 7 per group). Data were analyzed by rmANOVA followed by independent samples *t*-test. BL: baseline. ^∗^ *P* < 0.05; ^∗∗^ *P* < 0.01; ^∗∗∗^ *P* < 0.001 compared to 0 *μ*g/kg TNF-*α*.

To quantify the effects of systemic TNF-*α* administration on astrocyte activation over time, Gfap-luc mice were injected i.p. with different doses of TNF-*α* and bioluminescence was measured at specific time points. Factorial rmANOVA revealed a significant time × dose interaction (*F*(12,84) = 5.8; *P* < 0.001; *ε* = 0.53) for photons emitted per second in the brain ROI. Post hoc analysis demonstrated that, at 6 h after administration, a strong bioluminescent signal was present in the brain of TNF-*α*-injected mice (Figure 2). This signal was higher in mice injected with 250 *μ*g/kg TNF-*α* as compared to mice that received 63 *μ*g/kg TNF-*α*. Brain bioluminescence in mice treated with 63 *μ*g/kg TNF-*α* reached control levels at 24 h, while it took up to 72 h to normalize for animals injected with 250 *μ*g/kg TNF-*α*.

**Figure 3:**
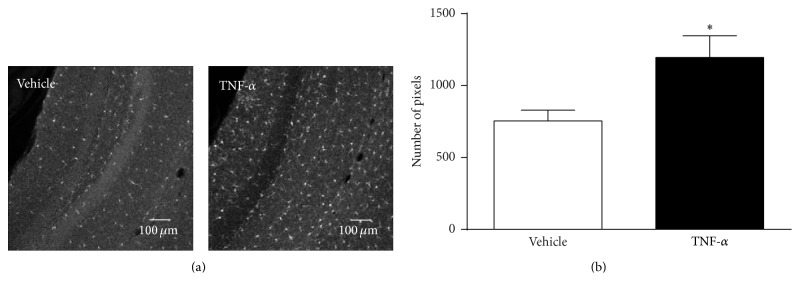
TNF-*α* increases Iba1 immunoreactivity in the dentate gyrus. TNF-*α* (250 *μ*g/kg, i.p.) caused a strong upregulation of the microglial activation marker Iba1 in the hippocampal dentate gyrus at 24 h after administration. Representative images (10x) (a) and image quantifications of *n* = 8 per group (b). Graph is plotted as mean + SEM. Data were analyzed by ANOVA followed by independent samples *t*-test. ^∗^ *P* < 0.05 compared to vehicle.

In order to confirm TNF-*α*-induced activation of glial cells by using a different technique and focusing on another cell type, immunohistochemistry was performed using a microglial activation marker. The expression of Iba1 was quantified in the hippocampal dentate gyrus at 24 h after systemic injection of vehicle or 250 *μ*g/kg TNF-*α*. This brain structure was chosen based on its association with stress and depression [37–39]. Pairwise comparison demonstrated that immunoreactivity against Iba1 in the dentate gyrus at 24 h was significantly higher in TNF-*α*-injected mice when compared to mice that received vehicle (*F*(1,13) = 7.3, *P* < 0.05) (Figure 3).

### 3.3. TNF-*α* Causes Sickness but No Depressive-Like Behavior

**Figure 4:**
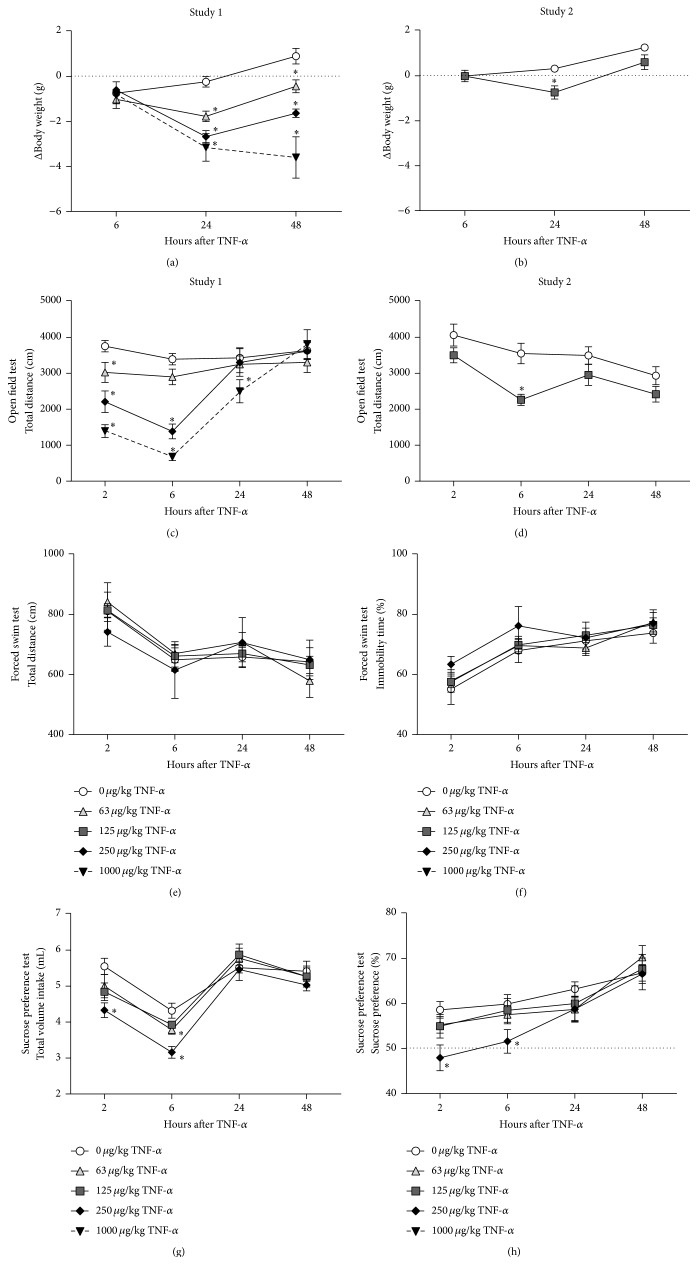
TNF-*α* causes sickness, but no clear depressive-like behavior. Systemic injection of TNF-*α* caused body weight loss ((a)-(b)), reduced locomotor activity in the open field test ((c)-(d)), and decreased total fluid intake in the sucrose preference test (g). Measures of behavioral despair in the forced swim test were not affected by administration of TNF-*α* ((e)-(f)). A high dose of TNF-*α* did decrease sucrose preference in the SPT (h) but this can be considered biologically irrelevant due to the overlapping time course of sickness. The dashed line in the sucrose preference test indicates the chance level (50%) for sucrose preference. Please note that the *y*-axis does not start at 0 for the forced swim and sucrose preference test data. Graphs are plotted as mean ± SEM (*n* = 10 per group, except *n* = 8 for 1000 *μ*g/kg TNF-*α*). Data were analyzed by rmANOVA followed by independent samples *t*-test. ^∗^ *P* < 0.05 compared to 0 *μ*g/kg TNF-*α*.

Sickness behavior in rodents is commonly evaluated by measuring changes in body weight and by assessing their locomotor activity in the OFT. Unfortunately, 2 out of 10 mice that were injected with 1000 *μ*g/kg TNF-*α* died within 24 h after administration. These subjects were removed from the analyses, resulting in a group size of *n* = 8 for this dose. rmANOVA showed a time × dose interaction for change in body weight (*F*(6,68) = 24.9; *P* < 0.001; *ε* = 0.95). Post hoc analysis demonstrated that there was a dose-dependent weight reduction at 24 h and 48 h after TNF-*α* administration (Figure 4(a)). Mice that were injected with 63 *μ*g/kg and 250 *μ*g/kg TNF-*α* started to gain weight at 48 h, while mice in the 1000 *μ*g/kg TNF-*α* group continued to lose weight.

A significant time × dose interaction was found for total distance travelled in the OFT (*F*(9,102) = 10.2; *P* < 0.001; *ε* = 0.70). At 2 h after systemic application, TNF-*α* reduced locomotor activity in a dose-dependent manner (Figure 4(c)). By 6 h, the total distance travelled by mice administered with 63 *μ*g/kg TNF-*α* had normalized to control levels, while it further declined in animals from the 250 *μ*g/kg and 1000 *μ*g/kg group. At 24 h, animals from the 250 *μ*g/kg group had recovered whereas this took up to 48 h for mice injected with 1000 *μ*g/kg TNF-*α*.

Based on the mortality rate of 20% in mice that received 1000 *μ*g/kg TNF-*α*, it was decided to take 250 *μ*g/kg TNF-*α* as the highest dose and to introduce a 125 *μ*g/kg TNF-*α* group in all of the behavioral experiments that followed. To test the effect of this additional dose on body weight and locomotor activity, a second, independent OFT was performed. rmANOVA showed a time × dose interaction for change in body weight (*F*(2,36) = 6.0; *P* < 0.05; *ε* = 0.74) and a main effect of time (*F*(3,54) = 14.2; *P* < 0.001; *ε* = 0.66) and dose (*F*(1,18) = 7.0; *P* < 0.05; *ε* = 0.66) for distance travelled in the OFT. Post hoc analysis revealed that the weight of mice injected with 125 *μ*g/kg TNF-*α* was reduced at 24 h, but not anymore at 48 h (Figure 4(b)). Moreover, systemic administration of 125 *μ*g/kg TNF-*α* decreased the distance travelled at 6 h, but not at any of the other time points measured (Figure 4(d)).

In the FST an animal is placed in a water-filled cylinder from which it cannot escape. Behavioral despair can be evaluated in this paradigm by quantifying duration of immobility, which can be confirmed by measuring the total distance the animal swims. rmANOVA revealed a significant effect of time, but not of dose, for total distance (*F*(3,108) = 20.4; *P* < 0.001; *ε* = 0.77) and immobility time (*F*(3,108) = 38.0; *P* < 0.001; *ε* = 0.75). Post hoc analysis showed that compared to the 2 h time point all animals swam less and remained immobile longer at 6 h, 24 h, and 48 h after TNF-*α* (Figures 4(e) and 4(f)). This happened independently of the TNF-*α* dose given and indicates habituation to the experimental procedure during retesting.

In the SPT an animal's preference for a sweetened solution versus water is measured. This paradigm allows evaluating sickness by assessing total volume intake while reductions in sucrose preference can be used as a measure for anhedonia, which is a key symptom of depression. rmANOVA demonstrated a significant effect of time (*F*(3,108) = 26.0; *P* < 0.001; *ε* = 0.98) and dose (*F*(3,36) = 4.0; *P* < 0.05; *ε* = 0.98) for total volume intake. Moreover, there was an effect of time (*F*(3,108) = 23.5; *P* < 0.001; *ε* = 0.96) and a trend for dose (*F*(3,36) = 2.6; *P* = 0.07; *ε* = 0.96) for sucrose preference. At 2 h and 6 h after administration, animals that were injected with 250 *μ*g/kg TNF-*α* drank significantly less than vehicle-treated controls, while mice that received 125 *μ*g/kg TNF-*α* only showed reduced fluid intake at 6 h (Figure 4(g)). Sucrose preference was lower at 2 h and 6 h in animals injected with 250 *μ*g/kg TNF-*α* but not at lower doses (Figure 4(h)).

**Figure 5:**
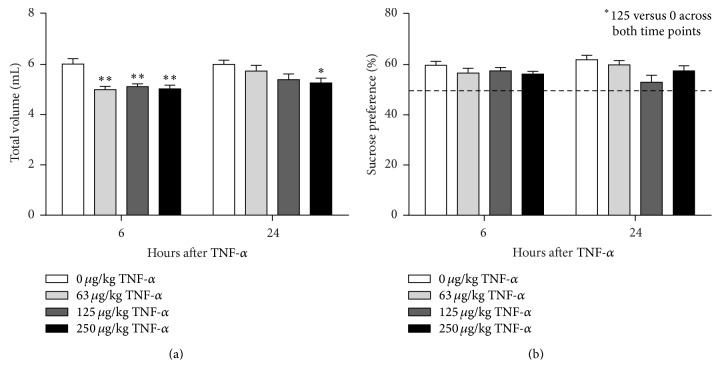
Peripheral TNF-*α* administration reduces total fluid intake (a) but does not induce clear anhedonia (b) in the sucrose preference test. Separate groups of naive animals were injected i.p. with TNF-*α* and tested in the SPT at either 6 h or 24 h. Dashed line indicates chance level for sucrose preference. Graphs are plotted as mean + SEM (*n* = 10 per group). Data were analyzed by ANOVA followed by independent samples *t*-test. ^∗^ *P* < 0.05; ^∗∗^ *P* < 0.01 compared to 0 *μ*g/kg TNF-*α* at the same time point.

All animals including the vehicle-injected controls showed reduced total volume intake at 6 h when compared to the other time points. This probably resulted from the fact that the fluid-deprived mice were allowed to drink at 2 h and hence were less thirsty at 6 h. To exclude that the effects of TNF-*α* were confounded by retesting the same animals over time, the SPT study was repeated for the 6 h and 24 h time points using separate groups of TNF-*α* challenged mice for each time point. In this second SPT study there was a main effect of time (*F*(1,72) = 5.7, *P* < 0.05) and dose (*F*(3,72) = 9.0, *P* < 0.001) for total volume intake and a main effect of dose (*F*(3,72) = 3.3, *P* < 0.05), but not time, for sucrose preference. Post hoc analysis revealed that using naive animals for each time point stabilized total volume intake in mice injected with vehicle (Figure 5(a)). At 6 h after administration, all TNF-*α*-treated mice drank less than their vehicle-injected controls. Volume intake at 24 h was only significantly reduced in mice administered with 250 *μ*g/kg TNF-*α*. Sucrose preference across both time points was reduced in mice that received 125 *μ*g/kg TNF-*α*, but not at any of the other doses (Figure 5(b)).

## 4. Discussion

A substantial set of literature data indicates a link between activation of the immune system and depression, at least in subpopulations of patients. Several clinical observations suggest that TNF-*α* is one of the key cytokines contributing to the development of inflammation-associated depression. In this series of experiments, we tested whether peripheral administration of TNF-*α* in mice is able to induce neuroinflammation as well as behavioral changes relevant to human depression.

TNF-*α* is a pleiotrophic cytokine that plays an important role in the early stages of inflammatory responses and in triggering the release of downstream immune molecules [40–42]. To assess the effect of peripheral TNF-*α* administration on immune activation in mice, we measured serum and brain levels of a selection of immune mediators. As expected, systemic injection of TNF-*α* caused a robust dose-dependent increase in circulating levels of TNF-*α*. Due to the fact that recombinant mouse TNF-*α* was administered, it was not possible to discriminate injected from endogenously produced TNF-*α*. Previous studies have shown that systemic injection of TNF-*α* upregulates cytokine gene expression in the liver [29, 43], thereby indicating that TNF-*α* is capable of eliciting a broad immunological response. In line with these findings, we found that peripheral TNF-*α* administration increased circulating levels of the proinflammatory immune mediators IL-6 and MCP-1. Moreover, the peak release of these factors was followed by an increase in the serum concentration of IL-10. This cytokine is a potent anti-inflammatory mediator that plays a role in attenuating inflammatory responses and suppressing the expression of proinflammatory cytokines [44]. Apart from MCP-1 levels in mice treated with the highest dose of TNF-*α*, the concentration of all cytokines had returned to baseline values at 24 h. This indicates that the inflammatory response to a single injection of TNF-*α* is short-lasting. Our cytokine data corroborates with findings from a recent study where systemic TNF-*α* was also reported to increase circulating levels of pro- and anti-inflammatory mediators [29]. However, not all of our findings are in line with those described by Skelly et al. In our study, for example, TNF-*α*-induced increases in IL-6 were of a higher magnitude than the ones previously described [29]. Moreover, in contrast to Skelly's data, we were not able to detect statistically significant increases in IL-1*β* levels. This was unexpected as TNF-*α* is known to induce the expression and release of IL-1*β* [29, 43]. These discrepancies may in part result from differences in experimental protocols, including the use of dissimilar recombinant TNF-*α*, mouse strains and gender, blood sampling methods, and potentially the sensitivity of the techniques used to quantify cytokine levels.

Several* in vitro* studies indicate that cross-regulation occurs between TNF-*α* and interferons [45–49]. However, results from these studies are often contradictory and the effect of TNF-*α* on interferon synthesis seems to depend on the inflammatory condition, the type of interferon (type I or type II), and the cell type studied [50]. Effects of TNF-*α* on IFN-*γ* levels* in vivo* are poorly described in literature. Our study did not detect a robust effect of TNF-*α* administration on IFN-*γ* levels in either serum or brain.

Cytokines from the periphery can pass the BBB through various mechanisms and access the brain [51]. TNF-*α* influences these processes in several ways. For example, TNF-*α* increases the permeability of the BBB [52], thereby facilitating the passage of relatively large molecules such as cytokines from the blood into the brain. Moreover, TNF-*α* stimulates the release of the chemokine MCP-1, which increases BBB permeability even further and subsequently drives the infiltration of leukocytes into the brain [53]. Accordingly, we found that brain levels of TNF-*α*, IL-6, and MCP-1 transiently increased in response to peripheral TNF-*α* administration. As we did not assess the integrity of the BBB, it is not clear whether these immune mediators entered the brain through a leaky BBB and/or if they were actively produced and released locally in the brain. It is possible that a fraction of the measured brain cytokine levels originated in the periphery. However, several studies described that bolus injected TNF-*α* is rapidly cleared and has a short half-life of up to 20 minutes [54–56]. These findings together with reports of cytokine gene expression in the brain following peripheral TNF-*α* administration [29] indicate that de novo transcription of these molecules does occur within the brain.

Besides elevated concentrations of proinflammatory cytokines, depressed patients frequently display increased circulating levels of MCP-1 [57, 58]. This chemokine is an important regulator of brain inflammation following a peripheral immune challenge [59, 60]. Additionally, MCP-1 has been suggested to act as a modulator of neuronal activity and neuroendocrine functions [61, 62]. In our study, peripheral TNF-*α* caused a robust release of MCP-1 both in serum and in whole brain tissue. As MCP-1 is preferentially expressed in the hippocampus and other neuroanatomical regions linked to depressive symptoms [62], it may play an important role in the development of inflammation-associated depression.

The neuroinflammatory response to peripheral TNF-*α* was further characterized using a transgenic mouse line that expresses luciferase under the transcriptional control of the Gfap promoter. GFAP is an intermediate filament protein that is predominantly expressed by astrocytes, and its expression is upregulated when astrocytes are activated [63]. These Gfap-luc mice thus allow noninvasive quantification of Gfap mRNA expression, as a marker of astrocyte activation, in living mice over time. We found that systemic administration of TNF-*α* caused a strong dose- and time-dependent activation of astrocytes. This TNF-*α*-induced astrocyte activation occurred after the peak release of proinflammatory cytokines and lasted for 2 days, thereby suggesting that the brain sequelae to a peripheral immune challenge may propagate in absence of the initial stimulus.

Although quantification of glial cell activation using bioluminescence imaging offers numerous advantages, this technique does not allow for spatial discrimination of specific brain regions. However, previous work has shown that neuroinflammatory responses to a peripheral immune challenge are brain region specific [64–66]. To confirm glial activation at a cellular level, focusing on another cell type and a specific brain area, we quantified the expression of Iba1 in the hippocampus. This brain structure is associated with depression and has previously been shown to display immune cell activation following a peripheral immune challenge [27, 67, 68]. In the brain, Iba1 is primarily expressed by microglia and its expression is upregulated upon microglial activation [69]. Consistent with measures of astrocyte activation in Gfap-luc mice, TNF-*α* injection increased Iba1 immunoreactivity in the hippocampus of FVB wild-type mice. This indicates that, in addition to astrocytes, microglia also show signs of activation following peripheral TNF-*α* administration.

Activated microglia are known to release proinflammatory cytokines, particularly TNF-*α* and IL-1*β*, but brain levels of these cytokines were not elevated at the time point at which we observed microglial activation. This may partly be explained by the fact that we quantified cytokine levels in the whole brain and not in specific brain regions. Moreover, assessing protein levels of cytokines in the brain is hampered by the limited sensitivity of available quantification techniques. This problem could be overcome by quantifying transcript expression using quantitative PCR, which is more sensitive approach compared to measuring protein levels of immune mediators in brain tissue. Cytokine as well as chemokine activity, however, is not only limited by gene expression, but also regulated at the posttranscriptional and posttranslational level [44, 70, 71]. Therefore, assessing protein levels of cytokines is suggested to be a more accurate indicator of cytokine activity [72]. Irrespective of differences in assay sensitivity, our data align with results from previous studies showing that cytokine expression in the hippocampus and hypothalamus was no longer elevated at 24 h after peripheral TNF-*α* administration [29].

To our knowledge, no study has systematically assessed the time course of sickness and depressive-like behavior following systemic TNF-*α* administration. After confirming that peripheral injection of TNF-*α* induces a central inflammatory response, we evaluated the time course of TNF-*α*-induced behavioral changes across a panel of assays commonly used to study sickness and depressive-like behavior in rodents. Our behavioral data demonstrate that TNF-*α* dose dependently induces sickness during the first 24 h after systemic administration. This could be seen as a decrease in body weight, reduced exploration in the OFT, and suppressed drinking in the SPT. In contrast to i.c.v. administration [30, 31], peripherally injected TNF-*α* did not affect measures of behavioral despair in the FST. Moreover, mild signs of anhedonia observed in the SPT overlapped with the time course of sickness and can therefore be considered biologically irrelevant. One limitation in our study is the within-subject design for the individual behavioral paradigms. This approach allowed reduction of animal numbers but also led to habituation of the mice to some of the experimental paradigms. Such habituation effects were observed in vehicle-injected control animals upon retesting in the FST (i.e., less swimming and longer immobility time) and in the SPT (i.e., less drinking at 6 h than at 2 h). To rule out that effects of TNF-*α* were missed because of habituation during retesting, the SPT study was repeated using separate groups of naive animals for the 6 h and 24 h time points. From this experiment it also became clear that peripheral TNF-*α* administration induced sickness, but not anhedonia. Taken together, the behavioral data indicate that acute systemic injection of TNF-*α* is not a reliable model to induce depressive-like behavior in mice. Based on the strong but short-lasting effects of TNF-*α* on neuroinflammation and behavior, it may be possible that prolonged or intermittent administration of TNF-*α*, leading to chronic upregulation of cytokines, offers a more valid approach to study depressive-like behavior in rodents. Such chronic TNF-*α* administration would mimic the human situation where inflammation-associated depression is believed to develop on a background of sustained, low-grade inflammation.

## 5. Conclusions

The present set of experiments using a variety of techniques and readouts showed that systemically administered TNF-*α* induced a strong but temporal release of immune mediators in the circulation and the brain. This release of inflammatory factors was followed by glial cell activation, as measured by astrocyte activation in the Gfap-luc mouse and increased Iba1 immunoreactivity in the hippocampus of FVB wild-type mice. Additionally, systemic administration of TNF-*α* led to a strong sickness response and mild signs of anhedonia. Due to the overlapping time course of these behavioral states it was not possible to unambiguously distinguish depressive-like behavior from sickness. Taken together, these results demonstrate that TNF-*α* in the periphery affects the central nervous system by inducing neuroinflammatory processes and behavioral changes.
